# GATA3 Regulates the Development and Functions of Innate Lymphoid Cell Subsets at Multiple Stages

**DOI:** 10.3389/fimmu.2017.01571

**Published:** 2017-11-14

**Authors:** Jinfang Zhu

**Affiliations:** ^1^Molecular and Cellular Immunoregulation Section, Laboratory of Immunology, National Institute of Allergy and Infectious Diseases, National Institutes of Health, Bethesda, MD, United States

**Keywords:** GATA3 transcription factor, innate lymphoid cells, development, cytokines, transcriptional activation

## Abstract

Innate lymphoid cells (ILCs) are regarded as the innate counterpart of effector CD4 T helper (Th) cells. Just as Th cells, ILCs are classified into distinct subsets based on their functions that are delivered mainly through effector cytokine production. Both ILCs and Th cells play critical roles in various protective immune responses and inflammatory diseases. Similar to Th cell differentiation, the development of ILC subsets depends on several master transcription factors, among which GATA3 is critical for the development and maintenance of type 2 ILCs (ILC2s). However, GATA3 is expressed by all ILC subsets and ILC progenitors, albeit at different levels. In a striking parallel with GATA3 function in T cell development and differentiation, GATA3 also has multiple functions in different ILCs at various stages. In this review, I will discuss how quantitative and dynamic expression of GATA3 regulates the development and functions of ILC subsets.

## Introduction

**Figure 1:**
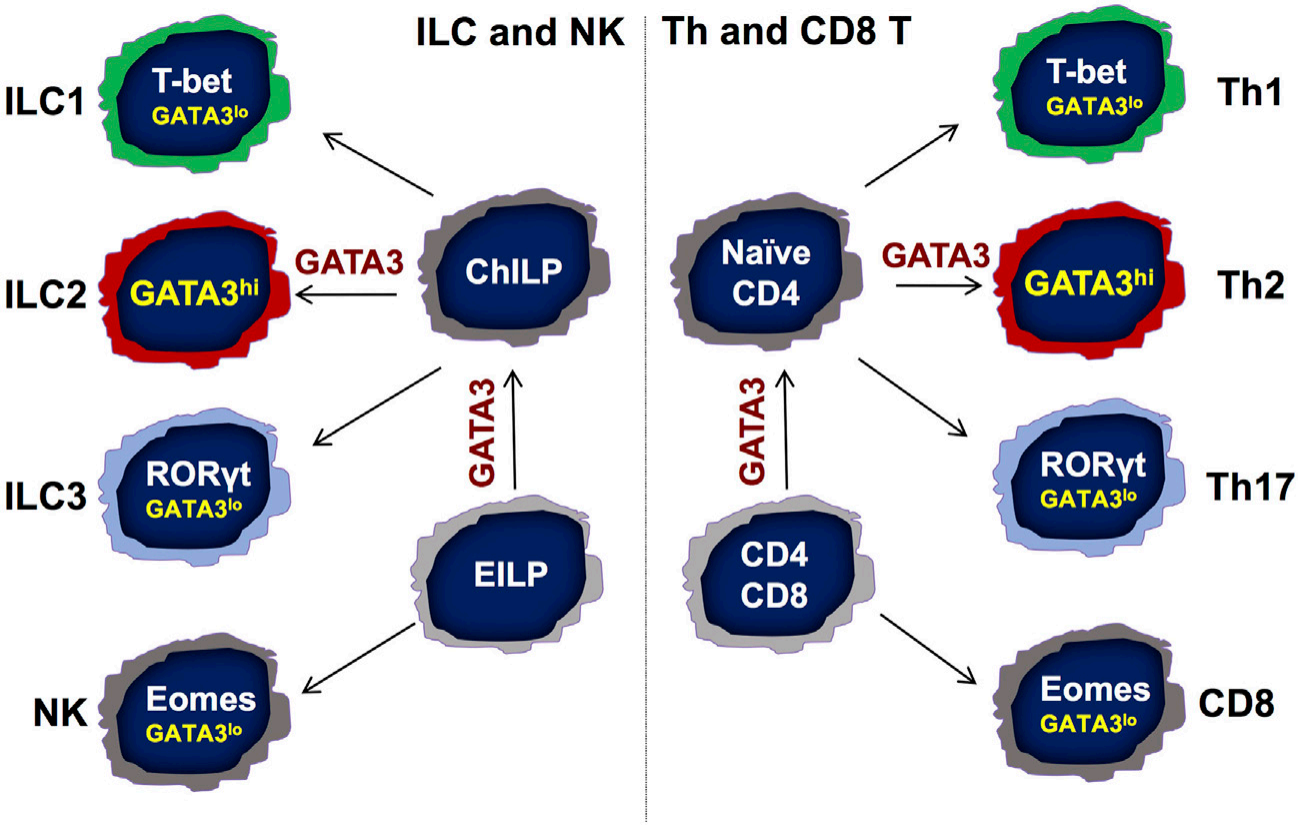
Symmetry between the development of adaptive and innate lymphocytes. There are following three major T helper (Th) subsets: Th1, Th2, and Th17 cells. Transcription factors T-bet, GATA3, and RORγt are the master regulators for the differentiation of Th1, Th2, and Th17 cells, respectively, from naïve CD4 T cells. Th2 cells express high levels of GATA3 that is critical for the development and functional maintenance of Th2 cells. The importance of low GATA3 expression in Th1 and Th17 cells is not clear. During T cell development, GATA3 is critical for the development of CD4 but not CD8 T cells in the thymus. Similarly, GATA3 is indispensable for the development of all IL-7Rα-expressing helper-like innate lymphoid cells (ILCs) but not natural killer (NK) cells. While GATA3 is critical for the functional maintenance of ILC2s, it is also required for the homeostasis, function, and further maturation of other ILCs, including ILC1s and ILC3s. Although GATA3 is not necessary for the development of NK and CD8 T cells, GATA3 expression at low levels contributes to the homeostasis and maturation of these cells. EILP, early ILC progenitor that expresses TCF7; ChILP, common helper-like ILC progenitor that expresses Id2.

Innate lymphoid cells (ILCs) are innate counterparts of CD4 T helper (Th) cells, which are considered professional cytokine-producing cells during adaptive immune responses. Based on their cytokine production and functions, just as Th cells, ILCs are divided into at least three major groups—group 1 ILC (ILC1), group 2 ILC (ILC2), and group 3 ILC (ILC3) subsets (1–4). ILC1s mainly produce IFN-γ; ILC2s produce IL-5 and IL-13; whereas ILC3s primarily produce IL-22. T-bet, GATA3, and RORγt are the master transcription factors for the development and functions of Th1, Th2, and Th17 (IL-17-producing Th) cells, respectively (5). Similarly, these master regulators are also critical for the development of ILC1s, ILC2s, and ILC3s, respectively (Figure 1). Natural killer (NK) cells are considered as the innate counterpart of CD8 T cells since both have cytolytic activity and depend on the transcription factor Eomes for their development.

Genome-wide analyses indicate that, in addition to the similarity in their transcriptomes, the epigenome of a given ILC subset is very similar to that of the Th counterpart especially at the cytokine loci (6, 7). Because of their similar capacity in producing effector cytokines (6–8), same class of ILC and Th subset may participate in specific type of immune responses (1, 6–11). Like Th1 cells, ILC1s may be involved in protective immunity against intracellular pathogens (12). Similar to Th2 cells, ILC2s are involved in type 2 immune responses to helminth infections (13–16). ILC3s and Th17 cells are important in dealing with infections of extracellular bacteria and fungi (17, 18). While ILC2s contribute to allergic inflammation (19–23), activation and expansion of ILC1s and ILC3s may induce certain types of autoimmunity (24). Interestingly, ILC activation may be sufficient to induce inflammation or to mount an effective immune response to infections in the absence of T cells under certain conditions (16, 25). Among the ILC3s, there is a distinct subset that expresses chemokine receptor CCR6 in mice (26). These mouse CCR6^+^ ILC3s represent lymphoid tissue inducer (LTi) or LTi-like cells that play a critical role in the development of lymphoid tissues including lymph nodes and Peyer’s patches (27, 28).

T helper and ILC subsets also differ from each other in many aspects. Because ILCs lack antigen receptors, they mainly respond to inflammatory cytokines to produce their own effector cytokines; IL-1 and IL-12 family cytokines including IL-1, IL-18, IL-33, and IL-23, as well as IL-25, are the major players in activating ILC subsets (1, 29, 30). Although Th cells mainly respond to antigens through activation of their T cell receptors, they can also respond to inflammatory cytokines in an antigen-independent manner, and these cytokines may play an important role in the maturation of Th cells (31, 32). Since antigen-specific Th cells need to be activated and expanded during an immune response while ILCs preexist in the tissue (33, 34), ILCs provide first line of host defense.

Another striking difference between Th cells and ILCs in their development is their dependence on cytokine signaling. While Th cell differentiation critically depends on cytokine-mediated activation of signal transducer and activator of transcription (STAT) family proteins (35), many STATs including STAT3, STAT4, and STAT6 are not required for ILC development (36–38). However, the response of mature ILCs to cytokines may still require the activation of STAT proteins (36–38). Nevertheless, STAT proteins that are important for cell proliferation and survival, especially STAT5 proteins (STAT5A and STAT5B), are important for the homeostasis of both Th cells and ILCs (39). In fact, ILCs are largely absent in common gamma chain (γc)-deficient animals and STAT5 is a major downstream molecule activated by cytokines that utilize γc (40).

Id2 plays an important role in ILC development but has a minimal effect on T cell development (12, 26, 41, 42). Some Id2-expressing progenitors in the bone marrow represent common helper-like ILC progenitors (12). On the other hand, the essential factor for T cell development Bcl11b only controls the development of ILC2s but not of other ILCs (26, 41, 43–47). Nevertheless, the development of ILC and Th cell subsets depends on many shared transcription factors such as TCF7, Tox, and GATA3 (8, 41, 48–50). Some TCF7-expressing cells in the bone marrow represent early ILC progenitor with a potential to become either NK cells or helper-like ILCs (48). Possibly because LEF1 (a TCF7 family member) (51) and Tox2 (52–54) are also expressed by ILCs and T cells, the blockage of ILC and T cell development in TCF7- or Tox-deficient animals is incomplete. On the other hand, since GATA3 is the only GATA protein expressed in T cells and ILCs, GATA3 is absolutely required for the development of T cells and ILCs (8, 55). In mature ILCs, while ILC2s express high levels of GATA3, all other ILCs express low levels of GATA3. In this review, I will discuss the functions of quantitative GATA3 expression in different ILC subsets and ILC progenitors in parallel with its important functions in the development and functions of T cell subsets.

## Critical Functions of GATA3 in ILC2s

ILC2s are enriched in the gut, lung, skin, and adipose tissues with few of them found in lymphoid tissues and in blood, and all the ILC2s express high levels of GATA3 (1, 13, 20). Like Th2 cells, ILC2s depend on GATA3 for their development. Even after ILC2s are fully mature, GATA3 remains important for their maintenance and functions, just as the essential role of GATA3 in differentiated Th2 cells (8, 56–64). In fact, GATA3 regulates a common set of important genes in both cell types, including *Il5, Il13, Areg, Il1rl1*, and *Ccr8*, which are well-known genes important for type 2 immune responses (8). This may explain identical functions of Th2 cells and ILC2s. Interestingly, the transcriptomes of ILC2s and Th2 cells harvested during helminth infection are remarkably similar (6).

Genome-wide analysis of GATA3 binding through ChIP-Seq shows that GATA3 binds to the *Il4/Il13* gene locus at multiple sites in both Th2 cells and ILC2s (65, 66). GATA3 also binds to the *Il5* and *Il13* promoters to induce their transcription (67, 68). Although the function of GATA3 in regulating epigenetic modifications at cytokine gene loci in ILC2s is not clear, GATA3 plays an important role in chromatin remodeling at the *Il4/Il13* locus in Th2 cells (65). GATA3 also directly binds to many genes that are involved in type 2 immune responses including *Il1rl1*, which encodes the IL-33 receptor subunit T1/ST2 (65, 66) and *Il17rb*, which encodes IL-25R (8). By regulating IL-33R and IL-25R expression, GATA3 is necessary for mature ILC2s to respond to inflammatory cytokines IL-33 and IL-25. ILC2s die quickly after GATA3 removal (8). It is possible that GATA3-deficient ILC2s fail to respond to cytokines. Alternatively, GATA3 may directly regulate cell proliferation and/or survival related genes in ILC2s (8).

## GATA3 Functions in ILC Progenitors

The functions of GATA3 in lymphocyte development and functional regulation are far beyond its critical role in Th2 cells and ILC2s, because all T cells and ILCs express different levels of GATA3 (66, 69, 70). We have previously reported that the development of IL-7Rα-expressing ILCs (or Th-like ILCs) but not of NK cells depends on GATA3 (8). Another report also indicates that ILC3 development requires GATA3 expression (50). This is consistent with the critical function of GATA3 during CD4 but not CD8 cell development (69, 71–73). It also supports the notion that ILC1s are developmentally distinct from conventional NK cells. Therefore, the development of innate (ILC) and adaptive (T cells) lymphocytes is highly symmetrical (Figure 1).

GATA3 promotes IL-7Rα expression in all T cells and ILCs, indicating that this may be a common mechanism through which GATA3 regulates lymphocyte homeostasis (66, 74). GATA3 expressed at low levels is sufficient to bind to the *Il7r* locus and regulates IL-7Rα expression in all ILCs and T lymphocytes (66, 74); the fact that the GATA3 binding pattern to the *Il7r* gene in ILC3s is identical to that in ILC2s and Th2 cells suggesting the existence of a high-affinity GATA3 binding site at the *Il7r* gene (66). However, GATA3-mediated IL-7Rα expression does not explain its critical role in the development of IL-7Rα-expressing ILCs because we have found that IL-7Rα transgene fails to rescue the ILC developmental defect in the absence of GATA3.

**Figure 2:**
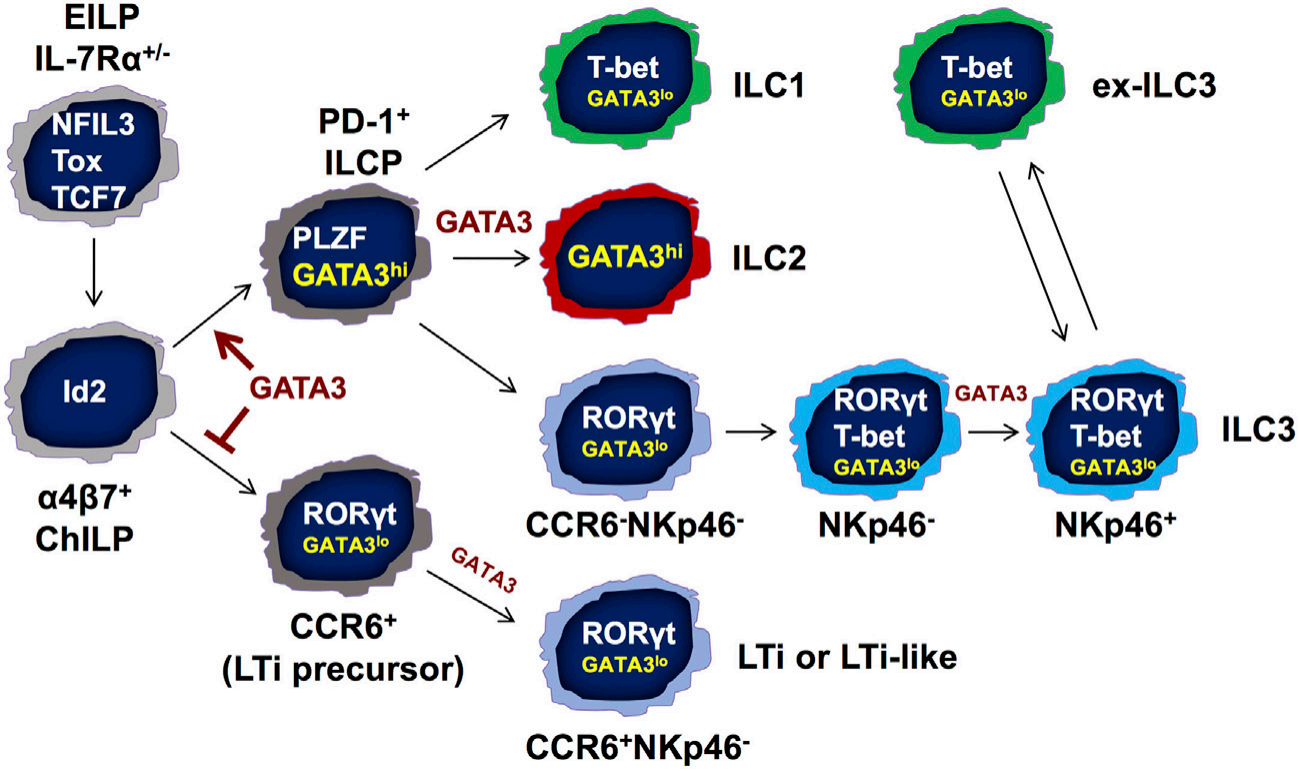
GATA3 regulates innate lymphoid cell (ILC) development and functions at multiple stages. GATA3 is expressed by all ILCs including lymphoid tissue inducer (LTi) cells and natural killer (NK) cells, albeit at different levels. GATA3 is required for the development of IL-7Rα-expressing ILCs but not NK cells as shown in Figure 1. At a possible developmental branch point for the bifurcation of PD-1^+^ PLZF-expressing ILC progenitors (ILCPs) and CCR6^+^ LTi progenitors, GATA3 is highly expressed in and absolutely required for the generation of “non-LTi” PLZF-expressing ILCPs. High level of GATA3 expression may suppress LTi developmental program. Nevertheless, GATA3 expression at low levels during the early stage of LTi cell development regulates LTi function. In mature ILCs, GATA3 is required for the functions and maintenance of ILC2s. GATA3 is also important for the homeostasis of ILC1s. Furthermore, the development of NKp46^+^ ILC3s that co-express T-bet and RORγt depends on low levels of GATA3 expression. Finally, GATA3 promotes IL-22 production in RORγt-expressing ILCs and regulates IL-7Rα expression in all ILC subsets. EILP, early ILC progenitor that expresses TCF7, NFIL3, and Tox; ChILP, common helper-like ILC progenitor that expresses Id2; ILCP, common ILC progenitor that expresses PLZF and GATA3.

It has been reported that the ILC1s, ILC2s, and non-LTi ILC3s are derived from ILC progenitors that express both PLZF (75) and PD-1 (76). These PLZF-expressing progenitors are known as common precursors to ILCs (ILCPs). However, CCR6^+^ LTi or LTi-like cells do not have a history of PLZF expression according to PLZF-fate-mapping experiments (75). We have previously reported that ILC numbers are dramatically reduced but not absent in the *Gata3*^fl/fl^-Vav-Cre conditional knockout mice (Vav-Cre is active at the hematopoietic stem cell stage) (8). Our recent results further demonstrate that LTi or LTi-like cells are the only ILCs that still remain in the GATA3 conditional knockout mice. By contrast, ILC1s, ILC2s, and CCR6^−^ ILC3s are undetectable in these mice. Interestingly, the PLZF-expressing ILCPs express high levels of GATA3 expression whereas the LTi or LTi-like progenitors express low levels of GATA3 in the bone marrow. Strikingly, the PLZF-expressing progenitors are completely lost in the bone marrow of *Gata3*^fl/fl^-Vav-Cre mice. The critical function of GATA3 for the generation of PLZF-expressing ILC progenitors is independent of cytokine signaling and/or IL-7Rα regulation since we have observed that these progenitors are present in normal numbers in γc-deficient animals. Thus, GATA3 is a critical regulator for the generation of PLZF-expressing ILC progenitors (Figure 2).

## GATA3 Functions in ILC3 Subsets

ILC3s are the major ILC population highly enriched in the gut (8, 18). RORγt, the master regulator of Th17 cells (77), is critical for the development and functions of ILC3s including both NKp46-expressing ILC3s and CCR6-expressing LTi or LTi-like cells (27). Although NKp46^+^ ILC3s may have some specialized functions in inflammation and gut homeostasis, by producing IL-22, NKp46^+^ ILC3s and CCR6^+^ ILC3s are functionally redundant in host defense (54, 78). NKp46-expressing ILC3s co-express T-bet and RORγt, whereas CCR6^−^ ILC3s in mice only express RORγt (66, 79, 80). Interestingly, both types of ILC3s express low levels of GATA3 (66).

T-bet and RORγt are critical for the development of NKp46^+^ ILC3s. During Th cell differentiation, transcription factors induced in one lineage can repress the expression and/or functions of transcription factors that are expressed by other lineages. For example, T-bet suppresses GATA3 expression as well as its function (81–83). In addition, T-bet and GATA3 bind to Th1- or Th2-specific genes at the same regions (83–85). T-bet also inhibits RORγt during T cell differentiation (86). NKp46^+^ ILC3s express slightly lower levels of RORγt than CCR6^+^ ILC3s, suggesting that T-bet may inhibit RORγt expression in these cells (80). Such inhibition may explain how “ILC1-like” “ex-ILC3s” can be generated through turning off RORγt expression (80). Interestingly, these “ex-ILC3s” may re-express RORγt and become ILC3s by IL-1/IL-23 stimulation (87).

Since the master transcription factors cross-regulate each other and they can be co-expressed at the single cell level, quantitative expression of different master regulators may alter the balance among these factors resulting in phenotypical changes. Indeed, haplo-insufficiency of the master regulators, including T-bet, GATA3, and RORγt has been reported (58, 66, 88). Interestingly, all three transcription factors, T-bet, GATA3, and RORγt, are expressed by NKp46^+^ ILC3s. More strikingly, low levels of GATA3 are also required for the development of NKp46^+^ ILC3s, and GATA3 regulates the balance between RORγt and T-bet (66). GATA3-deficient ILC3s express ~2-fold higher RORγt indicating that GATA3 inhibits RORγt expression in ILC3s. Correcting RORγt expression levels by breeding GATA3-deficient mice onto the *Rorc* heterozygous background restores the development of NKp46^+^ ILC3s, indicating that GATA3 regulates the balance between RORγt and T-bet during NKp46^+^ ILC3 development (Figure 2).

As mentioned earlier, GATA3 is not required for the development of LTi or LTi-like cells. However, these LTi cells are nonfunctional, since *Gata3*^fl/fl^-VavCre mice do not have lymph node structures (8). Single cell analysis of gene expression may address whether GATA3-deficient LTi cells fail to express some LTi-specific genes that determine LTi function. On the other hand, high levels of GATA3 may be inhibitory for the development of LTi progenitors since we have found that the expression levels of GATA3 are negatively associated with many LTi-specific genes at the single cell level. Furthermore, deletion of *Gata3* in NKp46^+^ ILC3s results in upregulation of CCR6^+^ ILC3-specific genes (66). Therefore, high levels of GATA3 expression at the PLZF-expressing progenitor stage are important for suppressing LTi lineage fate, and low expression of GATA3 in NKp46^+^ ILC3s is continuously required to maintain NKp46^+^ ILC3 cell identity by repressing the expression of LTi lineage-related genes.

GATA3 is also important for the optimal expression of *Il22* (66). Interestingly, GATA3 binds to the *Il22* promoter only in ILC3s but not ILC2s. Since GATA3 promotes IL-22 expression in both CCR6^+^ ILC3s and NKp46^+^ ILC3s, mice with ILC3-specific *Gata3* deletion mediated by RORγt-Cre are susceptible to *Citrobacter rodentium* infection. However, these mice develop normal lymph node structures. These results suggest that while GATA3 regulates LTi function at an early stage of their development, maintenance of LTi functions does not need continuous expression of GATA3 in LTi cells (66).

## GATA3 Functions in ILC1s and NK Cells

ILC1s including tissue-resident NK cells are enriched in the liver and T-bet is the master regulator for the development of ILC1s (12, 34). Similar to ILC3s, ILC1s also express low levels of GATA3 (12, 66). It has been reported that GATA3 is important for the maintenance of ILC1s (12). However, it is not known whether such GATA3 function is related to its effect on IL-7Rα expression in ILC1s. As discussed earlier, GATA3 is not necessary for the development of conventional NK cells (8, 89, 90). However, GATA3 is also expressed by NK cells, and they need GATA3 for their maturation and cytokine production (89).

## Regulation of GATA3 in ILCs and Their Progenitors

Since GATA3 plays important roles in different ILC subsets and progenitors, and its function is associated with its dynamic and quantitative expression, it is critical to understand signals that regulate GATA3 expression. During Th2 differentiation, IL-4-mediated STAT6 activation is the major driving force responsible for the upregulation of GATA3 expression. TCR-mediated signaling especially triggered by low dose of antigens can also upregulate GATA3 expression (91). However, ILCs do not express antigen receptors, and ILC2 development seems to be IL-4-STAT6 independent (37).

Notch signaling induces whereas TGFβ downregulates GATA3 expression (92, 93). These signaling pathways may be important in regulating GATA3 expression in different ILC subsets at different stages. Indeed, it has been reported that TCF7, which can be induced by Notch signaling, positively regulates GATA3 expression during early stages of ILC development (48, 59). Besides transcriptional regulation of *Gata3* gene expression, posttranscriptional and posttranslational regulations of GATA3 expression and functions should be also considered. For example, GATA3 activity is regulated by tyrosine kinase p38 in both T cells and ILCs (60, 94). Furthermore, GATA3 protein quickly disappears in ILC2s upon *Gata3* deletion indicating that there is an active mechanism for GATA3 protein degradation in these cells.

As another critical transcription factor involved in ILC development, Id2 may regulate GATA3 expression or *vice versa*. In the future, it is essential to understand the relationship between Id2 and GATA3. Because gene regulation mediated by GATA3 is highly cell type and stage specific, its induction may also depend on cell context. Identification of the cofactors for GATA3 and the gene regulatory networks in different cell types at various developmental stages is essential to understand the biology of GATA3 in ILCs.

## Concluding Remarks

T-bet, GATA3, and RORγt are the lineage-defining factors that are critical for the development and functions of ILC1s, ILC2s, and ILC3s, respectively. Besides its critical role in the development and maintenance of ILC2s, GATA3 is also indispensable for the generation of PLZF-expressing ILC progenitors that give rise to non-LTi ILCs. Furthermore, GATA3 regulates the acquisition of LTi cell function, ILC3 effector function, and ILC homeostasis (Figure 2).

T-bet, GATA3, and RORγt can be co-expressed at the single cell level resulting in heterogeneity and possible plasticity of ILC subsets. In particular, NKp46^+^ ILC3s express all three transcription factors. GATA3 expression in NKp46^+^ ILC3s cells, albeit at low levels, is important for regulating the balance between T-bet and RORγt. Because quantitative changes in GATA3 expression may result in qualitative developmental effects, a model with titratable GATA3 expression may be needed to study its dose effects on the development and functions of ILC subsets.

In addition to studying the dose effect of GATA3 in ILC development and functions, investigating cell type- and stage-specific GATA3 regulation and function will greatly enhance our knowledge on ILC development. It is important to identify cell type- or stage-specific enhancers within the 1Mb *Gata3* gene locus through epigenetic analyses. Future studies should also identify the similarities and differences between the actions of GATA3 in different cell types at various developmental stages, including both T cell and ILC lineages. Revealing cell type-specific gene regulation and epigenetic modifications mediated by GATA3 as well as cell type-specific binding patterns of GATA3 at a genome level will help us understand detail mechanisms. Finding GATA3 partners in different cell types, either by using co-immunoprecipitation followed by mass spectrometry or by genome-wide screening through bimolecular fluorescence complementation methods, may explain cell type-specific gene regulation mediated by GATA3.

Besides GATA3, many other transcription factors such as Id2, TCF7, NFIL3, Tox, and Runx proteins are also involved in ILC development (8, 41, 42, 48, 49, 95, 96). Further investigation on the relationship between GATA3 and other important transcription factors that are involved in ILC development and functions, including studies at a single cell level, may yield a deeper understanding of the ILC biology.

## Author Contributions

The author confirms being the sole contributor of this work and approved it for publication.

## Conflict of Interest Statement

The author declares that the research was conducted in the absence of any commercial or financial relationships that could be construed as a potential conflict of interest.
